# 2161. Biofilm and Aggregate Formation as an Efficient Strategy Used by *C. parapsilosis* to Persist Echinocandin Treatment and Evade the Innate Immune Cells

**DOI:** 10.1093/ofid/ofad500.1784

**Published:** 2023-11-27

**Authors:** Farnaz Daneshnia, Daniel Floyd, Süleyha Hilmioğlu-Polat, Macit Ilkit, Agostinho Carvalho, Carol Munro, David Perlin, Toni Gabaldon, Clarissa Nobile, Amir Arastehfar, Michael Mansour

**Affiliations:** Massachusetts General Hospital, Boston, Massachusetts; Massachusetts General Hospital, Boston, Massachusetts; Ege University, Izmir, Izmir, Turkey; University of Çukurova, Adana, Adana, Turkey; UMinho, Braga, Braga, Portugal; University of Aberdeen, Aberdeen, Scotland, United Kingdom; Hackensack Meridian School of Medicine, Nutley, New Jersey; Comparative Genomics group, Barcelona, Catalonia, Spain; University of California Merced, Merced, California; Massachusetts General Hospital, Boston, Massachusetts; Massachusetts General Hospital, Boston, Massachusetts

## Abstract

**Background:**

Biofilm and aggregate formation are thought to be critical strategies employed by fungal pathogens, such as *Candida auris* and *Candida parapsilosis*, to effectively colonize biotic and abiotic surfaces as well as to cause clonal outbreaks. Herein, we identified an immunocompetent patient experiencing persistent candidemia due to *C. parapsilosis* while under micafungin treatment. Four *C. parapsilosis* isolates were collected during the course of treatment, the initial isolate from the central venous catheter and the latter isolates from blood samples. We aimed to investigate microbiological attributes leading to immune evasion and micafungin therapeutic failure.

**Methods:**

Whole genome sequencing (WGS) was employed to assess the genetic relatedness of the isolates. Detailed biofilm and aggregate formations were evaluated using confocal microscopy and cell wall staining and scanning electron microscopy was employed to assess cell wall changes. Interaction with primary neutrophils and macrophages and fungal burden in various organs of mice systemically infected with our isolates was carried out to assess the fitness.

**Results:**

Whole-genome sequencing found isolates clonal. Interestingly, the latter isolates formed larger aggregates and thicker biofilm and a high concentration of micafungin was less effective against their biofilm structures. Detailed cell wall analysis found a concomitant incremental increase in mannan and a lower β-glucan and chitin of the latter isolates. Additionally, the latter isolates were less effectively phagocytosed by primary human macrophages and neutrophils, and macrophages infected with the latter isolates secreted significantly lesser proinflammatory cytokines, including TNFα and IL1β. Finally, mice infected with the latter isolates had a significantly higher burden in the kidney, liver, and spleen when compared to the initial isolate. Genomic changes underlying such morphological changes are under investigation, which will be used for transformation and functional studies.

**Conclusion:**

Altogether, our study highlights that under certain conditions biofilm and aggregate formations can be employed by *C. parapsilosis* to effectively persist echinocandin treatment and evade the immune system.

**Disclosures:**

**Michael Mansour, MD, PhD**, Thermofisher: Grant/Research Support

